# Cathodal transcranial direct current stimulation over posterior parietal cortex enhances distinct aspects of visual working memory

**DOI:** 10.1016/j.neuropsychologia.2016.04.028

**Published:** 2016-07-01

**Authors:** Klaartje Heinen, Laura Sagliano, Michela Candini, Masud Husain, Marinella Cappelletti, Nahid Zokaei

**Affiliations:** a Institute of Cognitive Neuroscience, University College London, London, England; b Department of Experimental Psychology, University of Oxford, Oxford, England; c Department of Psychology, Second University of Naples, Caserta, Italy; d Department of Psychology, University of Bologna, Bologna, Italy; e Department of Psychology, Goldsmiths, University of London, London, England; f Oxford Centre for Human Brain Activity, Department of Psychiatry, University of Oxford, Oxford, England

**Keywords:** Transcranial direct current stimulation, Parietal cortex, Polarity, Visual working memory, Precision, Error source

## Abstract

In this study, we investigated the effects of tDCS over the posterior parietal cortex (PPC) during a visual working memory (WM) task, which probes different sources of response error underlying the precision of WM recall. In two separate experiments, we demonstrated that tDCS enhanced WM precision when applied bilaterally over the PPC, independent of electrode configuration. In a third experiment, we demonstrated with unilateral electrode configuration over the right PPC, that only cathodal tDCS enhanced WM precision and only when baseline performance was low. Looking at the effects on underlying sources of error, we found that cathodal stimulation enhanced the probability of correct target response across all participants by reducing feature-misbinding. Only for low-baseline performers, cathodal stimulation also reduced variability of recall. We conclude that cathodal- but not anodal tDCS can improve WM precision by preventing feature-misbinding and hereby enhancing attentional selection. For low-baseline performers, cathodal tDCS also protects the memory trace. Furthermore, stimulation over bilateral PPC is more potent than unilateral cathodal tDCS in enhancing general WM precision.

## Highlights

• Despite multiple studies, reported effects of tDCS on cognitive processes have remained variable.
• We employ a WM task that probes different underlying sources of error to test effect of tDCS separately.
• tDCS applied bilaterally to the PPC boosts WM precision.
• Unilateral stimulation indicates this is caused by cathodal- rather than anodal stimulation.
• Improvement relies particularly on enhanced selective attention as well as memory trace protection.

## 1 Introduction

Non-invasive brain stimulation techniques such as transcranial direct current stimulation (tDCS) can be applied to interfere with ongoing neural activity and thereby obtain insight into underlying *causal* mechanisms of cognitive processes (de Graaf and Sack, 2014). Moreover, recent years have seen increased interest in its potential as a therapeutic method to enhance cognitive performance (Kuo and Nitsche, 2012, Sandrini and Cohen, 2013). The main effect of tDCS is thought to be the enhancement or suppression of local neural activity through respectively depolarising (anodal) or hyperpolarising (cathodal) the membrane potential (Nitsche et al., 2008). Consistent effects have been reported with tDCS applied to the motor cortex: anodal stimulation facilitates motor function, whereas cathodal stimulation has a suppressive effect (Csifcsak et al., 2009, Fregni et al., 2006, Furubayashi et al., 2008, Jeffery et al., 2007, Lang et al., 2004a, Nitsche et al., 2008, Nitsche and Paulus, 2000, Stagg et al., 2009). However, the effects of tDCS and polarity on higher-order cognitive functions are far less clear and have yielded variable results (see (Horvath et al., 2015, Jacobson et al., 2012) for a review).

One reason for the observed variability of effects might be that cognitive processes rely on different underlying sub-mechanisms that may be differently affected by tDCS. Such a possibility highlights the need for sophisticated behavioural paradigms that can probe separate underlying mechanisms and permit testing how these are affected by different types of stimulation.

Working memory (WM) is crucial for many higher-order cognitive functions (Goodale et al., 2004) and is associated with activity in the dorsolateral prefrontal cortex (Curtis and D’Esposito, 2003, Postle, 2006) and the posterior parietal cortex (PPC) (Todd and Marois, 2005, Vogel et al., 2005, Xu and Chun, 2006). Only a few studies have investigated the effects of tDCS over PPC on visual WM and yielded variable results. Performance on a change-detection WM task was enhanced after right PPC was stimulated with anodal tDCS, but only for low baseline-performers (Tseng et al., 2012). Another group, who tested the impact of tDCS over the right IPS, demonstrated that anodal stimulation impaired-, while cathodal stimulation enhanced WM capacity (Heimrath et al., 2012). In a third study, cathodal stimulation selectively impaired WM on recognition- but not on retrieval trials and no effects of anodal stimulation were observed (Berryhill et al., 2010). Finally, in a paradigm that tested effects of parietal tDCS on attentional mechanisms, it was found that only cathodal tDCS enhanced attentional selection (Moos et al., 2012).

The limit of visual WM performance in these studies was tested in a binary, two-alternative forced choice manner, where observers were asked to make a yes/no response. However, such measures might not be sensitive indices of WM (Zokaei et al., 2014) and they do not permit dissection of the different *sources of error* influencing recall (Ma et al., 2014). To investigate how tDCS affects distinct sub-mechanisms underlying visual WM, we employed an experimental method of measuring visual WM, which, unlike binary measures, examines the precision with which items are recalled (Bays et al., 2011). This technique is referred to as a delayed reproduction task: participants reproduce from memory the feature of a probed item using a continuous, analogue response space. The question here is not whether someone remembers an item or not, but rather how well – or precisely – they recall it.

During the employed task, orientations of four simultaneously presented coloured bars have to be remembered over a delay, after which the orientation of one of the bars is cued – by its colour – for recall. Participants have to adjust the orientation of the cued bar until it matched the orientation held in memory. Importantly, this paradigm provides a means to assess general WM precision as well as to dissect out sources of error contributing to the pattern of performance, by using a probabilistic model. In this approach, error can arise from different sources (Bays, et al., 2011). Firstly, it can be due to an increase in *variability* of the memory for the probed (or target) orientation, i.e. noisiness of memory for that item, which is an indication how well the memory trace was ‘protected’ during the retention period. Secondly, it can be due to an increase in proportion of responding to the orientation of one of the other orientations held in memory. These are trials where items that were not probed – non-target items – systematically corrupt memory by biasing recall such, that observers report the orientation of a bar of a different colour to the target item. In other words, they misbind the colour of the probed item to the orientation of one of the other items in memory, which is a measure of impaired selective attention. Finally, an increase in proportion of responding in a *random* fashion independent of any orientation in memory can contribute to error in performance. This can be due to different factors such as inattention, distraction, compliance with the task etc. By applying a probabilistic model (previously used by (Bays et al., 2011)) we are able to deconstruct sources of error in our working memory task.

The aim of the current experiment was to test how different applications of tDCS over the PPC (bilateral and unilateral anodal- and cathodal stimulation), affected these different aspects of visual WM. Based on earlier studies, we expected that tDCS would influence WM performance (Berryhill et al., 2010, Heimrath et al., 2012, Tseng et al., 2012), but that this effect might depend on the configuration of the anodal- and cathodal electrodes. In contrast to the change detection task used in earlier studies, the underlying neural correlates of the different possible sources of error in the current WM-task have not been clearly established. However, based on earlier findings, we expected that attentional mechanisms subserving WM may be positively affected by cathodal stimulation over the right PPC (Moos et al., 2012). We held less clear expectations how either polarity over the PPC may affect the protection of the memory trace. In Experiment I, we applied bilateral stimulation, with the anodal electrode placed on the right- and the cathodal on the left hemisphere, while in Experiment II participants received stimulation with reversed polarity. Participants in both experiments received Sham stimulation in a separate session as control. In a third experiment we only stimulated right PPC, with either anodal, cathodal or Sham stimulation, while a reference electrode was placed on the opposite arm.

## 2 Material and methods

### 2.1 Participants

Overall fifty-one right-handed healthy volunteers took part in the study. Following screening for any contra-indications to tDCS, all provided informed consent in accord with local ethics clearance.

All had normal or corrected-to-normal vision. Sixteen volunteers (6 male 10 female; age 19–37 years) took part in Experiment I, another 16 (4 male 12 female; age 19–38 years) in Experiment II and 19 in Experiment III (10 males; age range 19–33 years). Outlier analysis on Precision- and/or Bias-scores (>2 STDEV) excluded 2 participants from Experiment II and 3 participants from Experiment III.

### 2.2 Experimental procedure

**Fig. 1:**
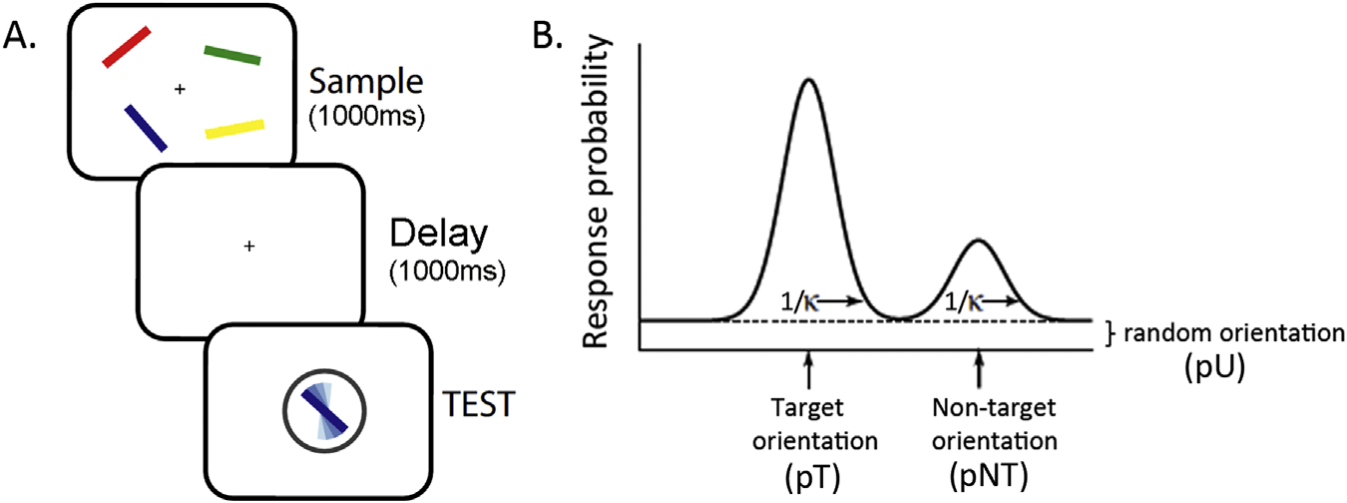
Schematic representation of the experimental paradigm. Four coloured bars, each with different orientation were presented during the initial display for 1000 ms. Following a delay of 1000 ms, participants were probed by a coloured (test) bar to reproduce the orientation of one of the four originally presented bars with corresponding colour, by manipulating the (test) bar with a response mouse.

A schematic representation of the experimental task is depicted in Fig. 1. A 1000 ms central fixation cross was presented at the beginning of each trial, followed by a display containing 4 coloured bars (2°x0.3° of visual angle) presented on a grey background on a 21 in. CRT monitor at a viewing distance of 60 cm. The bars were presented on an invisible circle with radius of 6° of visual angle. Orientation of the four bars was chosen randomly (0–180°) and their colours were selected by random permutation of six easily distinguishable colours. The distribution of the bars was pseudo-randomised so that 2 bars were presented in the left and 2 bars in the right hemifield. The display was presented for 1000 ms followed by a blank (grey) screen for 1000 ms. One of the bars was then centrally probed by colour with a random orientation. Participants had to match the remembered orientation of the same-coloured bar in the memory display (target) by rotating the probe using the response mouse. Participants did not know beforehand which of the four bars would be probed.

Each experimental session consisted of two parts. During the first part the task was performed without tDCS (pre-stimulation) and during the second part tDCS was applied during the task. Each part comprised three blocks each with 36 trials (108 trials in total), lasting ca 15 min. Targets would appear in the left or right hemifield with 50% probability.

### 2.3 tDCS

Direct current was transferred by a saline-soaked pair of surface sponge electrodes (6.5×4.5 cm) and delivered by a battery-driven, constant current stimulator (Magstim, Carmathenshire, UK). In Experiment I the anodal electrode was placed over P4 and the cathodal electrode over P3 according to the 10–20 International system for EEG electrode placement. On a separate day and counterbalanced across participants, non-effective Sham stimulation was applied (see below). In Experiment II the electrode polarity was reversed and non-effective Sham stimulation was again applied on a separate day (counterbalanced). In Experiment III tDCS was applied unilaterally, with either effective anodal- or cathodal electrode placed over the right PPC while the other electrode was placed on the contralateral left arm or with non-effective Sham (on separate days and counterbalanced across participants). A constant current of 1.5-mA intensity was applied, starting 5 min preceding and continuing for another 15 min during the task. Participants could feel the stimulation as an itching sensation at the beginning of the stimulation. In the Sham condition, the stimulator was switched off after 30 s so that any initial itchy sensation felt was in common with real stimulation.

### 2.4 Eye tracking

In all experiments, eye position was acquired with a video eye tracker at 500 Hz (EyeLink 1000, SR Research) throughout the task. Data were analysed for saccades (>150 ms duration) during the 1000 ms of the trial during which the stimulus display was presented. Participants maintained fixation in >90% of the trials. No effects of tDCS on saccades was observed (p>.4). No trials were excluded.

### 2.5 Analysis

For each trial, a measure of error was obtained by calculating the angular deviation between the orientation reported by the subject and the correct orientation of the target bar in the preceding display. Recall precision was defined as the reciprocal of standard deviation of error in response for each condition.

To quantify the variability of recall and the contribution of different sources of error in each experiment, we applied a probabilistic model introduced previously by (Bays et al., 2011). This model attributes errors on the reproduction task to three sources. In tasks similar to the one employed here, error can arise due to increased variability in memory for target feature, here orientation. Alternatively, error can arise as a result of misreporting one of the other non-target orientations in the sequence. These are trials where other items in memory systematically bias target memory. Further, responding with a random orientation not related to any of the items in the sequence (i.e. guesses) can result in error in response. This model is described as follows:

$$p(\hat{\theta })=a\phi_{K}(\hat{\theta }-\theta )+\beta \frac{1}{m}\sum_{i}^{m}\phi_{K}(\hat{\theta }-\varphi_{i})+\gamma \frac{1}{2\pi }$$

where θ is the true orientation of the target item, $\hat{\theta }$ the orientation reported by the subject, and Φ_κ_ is the von Mises distribution (the circular analogue of the Gaussian distribution) with mean of zero and concentration parameter κ. Concentration parameter κ reflects the variability of recall of the target feature- higher κ corresponds to lower variability. The probability of reporting the correct target item (pT) is given by α. The probability of misreporting a non-target item (pNT) is given by β, and {ϕ_1_, ϕ_2_, …ϕ_m_} are the orientations of the *m* non-target items. The probability of responding randomly (pU) is given by γ=1-α-β. Maximum likelihood estimates (Myung et al., 2013) of the parameters κ, α, β and γ were obtained separately for each subject and experimental condition (stimulated site, pre/during stimulation, target hemifield) using an expectation–maximization algorithm (MATLAB code available at http://www.sobell.ion.ucl.ac.uk/pbays/code/JV10/) and effects were tested by ANOVA and *t* tests. A schematic representation of these model components is given in Fig. 1(b).

## 3 Results

In the first two experiments, we tested the effects of tDCS on WM precision and underlying sources of error, when applied bilaterally over the posterior parietal cortex (PPC, electrode equivalent P3 and P4). Based on earlier findings, we expected that tDCS over (right) PPC would influence WM performance (Berryhill et al., 2010, Heimrath et al., 2012, Tseng et al., 2012), but that effects might depend on the configuration of the anodal- and cathodal electrodes. We therefore placed the anodal electrode over right- and the cathodal electrode over left PPC in Experiment I, with a reversed polarity configuration in Experiment II. Participants would perform the WM precision task without tDCS during the first session and with tDCS during a subsequent second session with a short break in between. During the task, participants were probed by colour to reproduce the orientation of one out of four presented coloured bars (see Fig. 1 for a schematic representation). WM precision was defined as the reciprocal of standard deviation of error in response for each condition. To estimate the effects on the different underlying sources of error separately, a probabilistic model (Bays et al., 2011) was subsequently applied to the data, yielding values for: (1) **kappa (κ):** concentration measure reflecting a circular Gaussian *variability – or noisiness – of recall* of orientation. This could be seen as a measure how well the memory trace was protected during the retention period (2) **pNT:** probability of responding to the orientation of one of the non-targets (*misbinding errors*), a measure for selective attention. (3) **pU:** probability of responding randomly (i.e. guesses). A change in (2) and (3) would be reflected in an overall change in the probability of responding correctly to the orientation of the target which is referred to as **pT** (see Fig. 1).

Effects of stimulation on these parameters were tested with an ANOVA including the within-participant factors of tDCS (pre-stim and stim), type of stimulation (tDCS vs. Sham) and target hemifield.

**Fig. 2:**
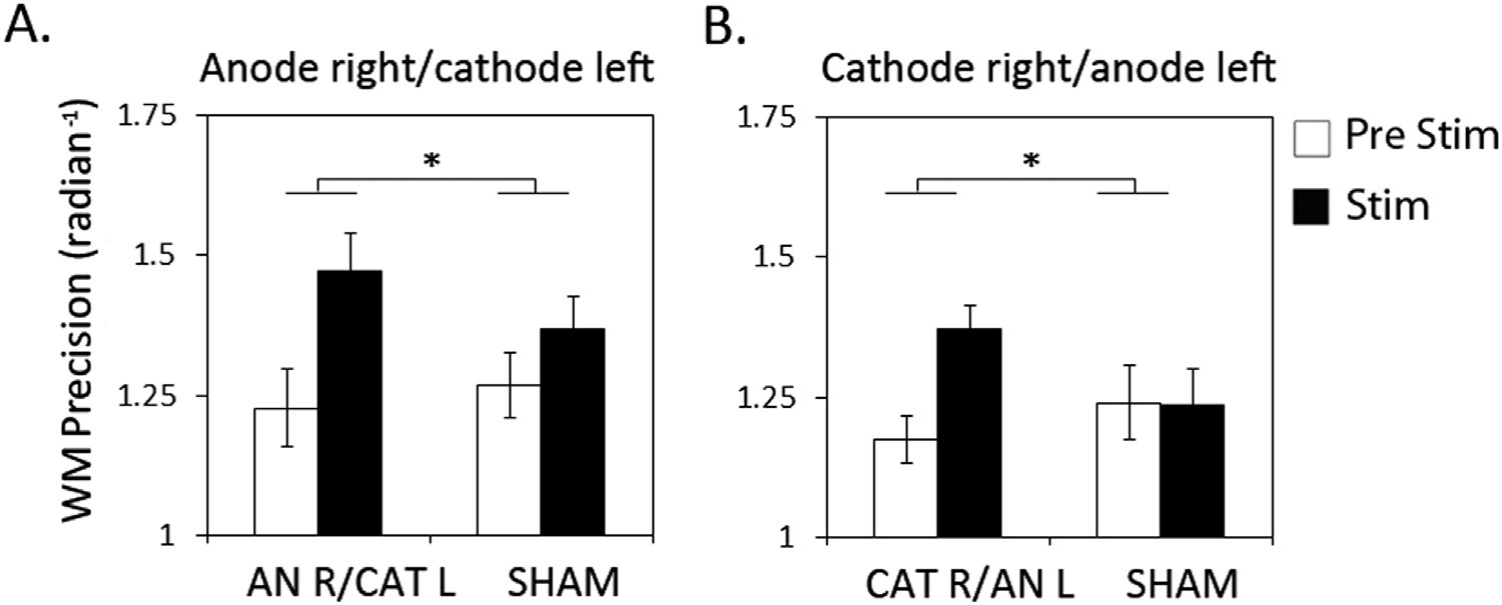
Displayed are WM precision scores pre-stimulation (pre-stim baseline) and during stimulation with bilateral tDCS over PPC. An enhancement in WM precision relative to baseline, greater than during Sham stimulation, is observed both with A) anodal electrode over right- and cathodal electrode over left PPC (Experiment I) and with B) cathodal electrode over right- and anodal electrode over left PPC (Experiment II).

**Fig. 3:**
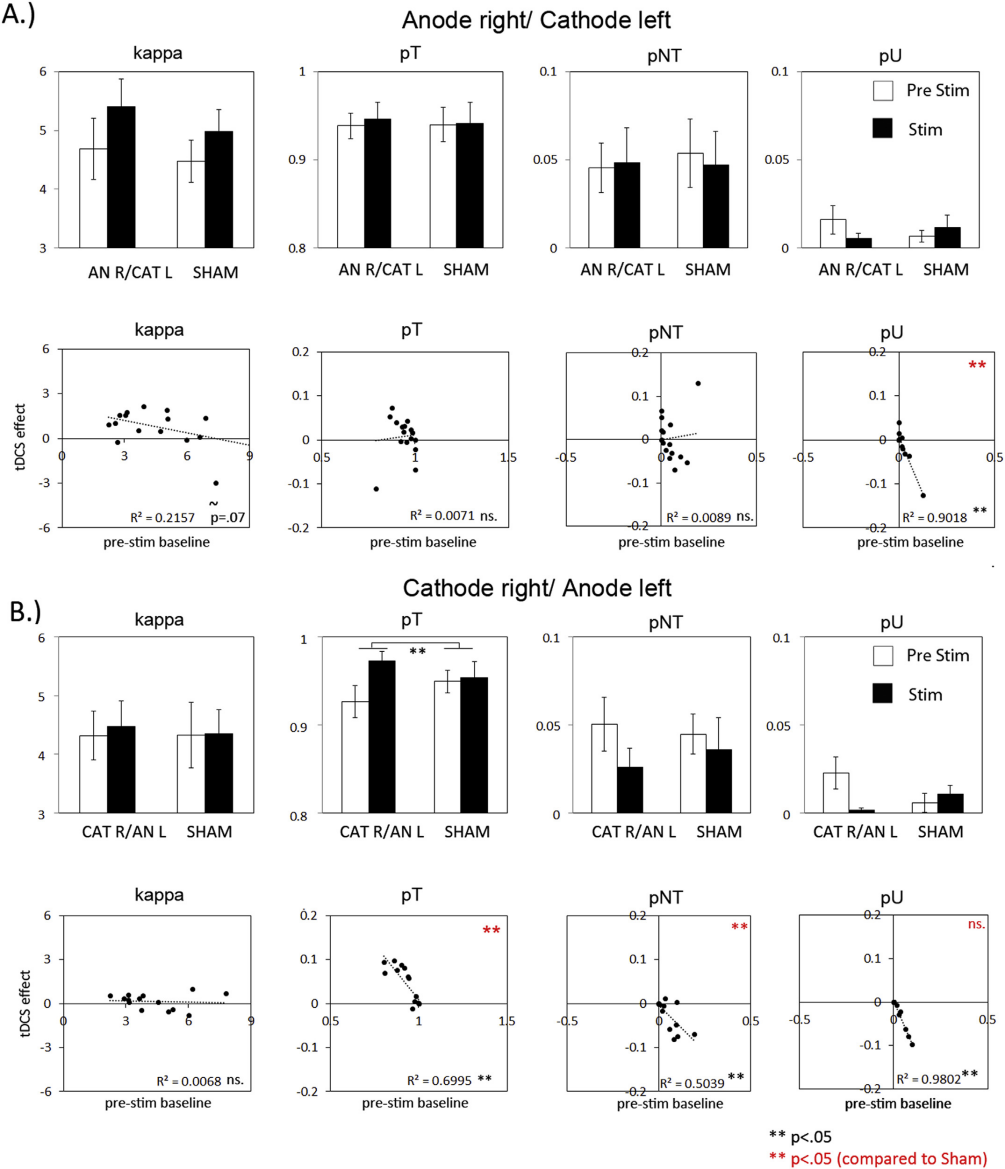
Effects of bilateral tDCS over PPC on different sources of error underlying WM precision: variability of response (kappa), probability of correct target response (pT) and probability of error through misbinding (pNT) or random response (pU). No differences were found for any of the parameters in Experiment I (A, first row, plot 1–4). However, a negative correlation between baseline performance and tDCS-effect for pU (A, second row, plot 4) indicates a suppressive effect of tDCS on random errors, if these errors were highly prevalent during baseline. In Experiment II tDCS enhanced pT across participants (B, first row second plot). A negative correlation with baseline performance, as observed both for pT and pNT, indicates that the enhanced proportion correct response (pT) is due to a decrease in misbinding errors (pNT), for low-baseline performers (B, second row, plot 2 and 3). Note: the observed negative correlation for pU, did not differ significantly from Sham (plot 4).

With the electrode configuration in Experiment I (anodal on right- and cathodal on left hemisphere), we observed a significant increase in WM precision when comparing performance during tDCS (stim) with that during the pre-stimulation (pre-stim) session, which was significantly greater than that during Sham stimulation (tDCS×tDCS type F(1,15)=6.6 p=0.022; see Fig. 2(A)). This effect did not differ across hemifields (tDCS×tDCS type×hemifield p>.75 ns). Application of the probabilistic model revealed (despite trends) no significant main effect or interaction of tDCS on any of the parameters (see Fig. 3(A)). However, a negative correlation was observed between the tDCS-effect (stim minus pre-stim) and baseline scores (pre-stim) for pU (see Fig. 3(A) second row, last plot), indicating that random errors were more reduced by tDCS, if highly prevalent during baseline performance. This correlation was significantly different when compared to Sham (Steiger's comparison between correlations Z=−3.2 p<.05). No such correlations were found for any of the other parameters.

With the *reverse* electrode configuration in Experiment II (cathode on right- and anode on left hemisphere), we also observed a significant increase on overall WM precision, again significantly greater compared to Sham (see Fig. 2(B): tDCS×tDCS type F(1,13)=7.3 *p*=0.02) and across hemifields (tDCS×tDCS type×hemifield *p*>.8 ns.). Estimating the sources of error, revealed a positive effect of tDCS on probability of correct target response (pT) (see Fig. 3(B) first row, second plot; tDCS×tDCS type F(1,13)=4.6 *p*=0.05) across hemifields (tDCS×tDCS type×hemifield: p>.3 ns.). Despite trends, there were no significant effects on pNT, pU or the variability of recall (kappa). When correlating the tDCS-effect on these variables with the scores during baseline (pre-stim) however, significant negative correlations were observed both for pT and pNT (see Fig. 3(B) second row, plot 2 and 3). These correlations were not observed for Sham (Steiger's comparison between correlations Z=−3.2 (pT) and Z=−3.0 (pNT) p<.05). We also observed a strong negative correlation for pU, however, this was not significantly different from Sham. This indicates that tDCS improved proportion of correct scores (pT) and that this was due to a reduction in misbinding error (pNT), which was observed only for low-baseline performers.

**Fig. 4:**
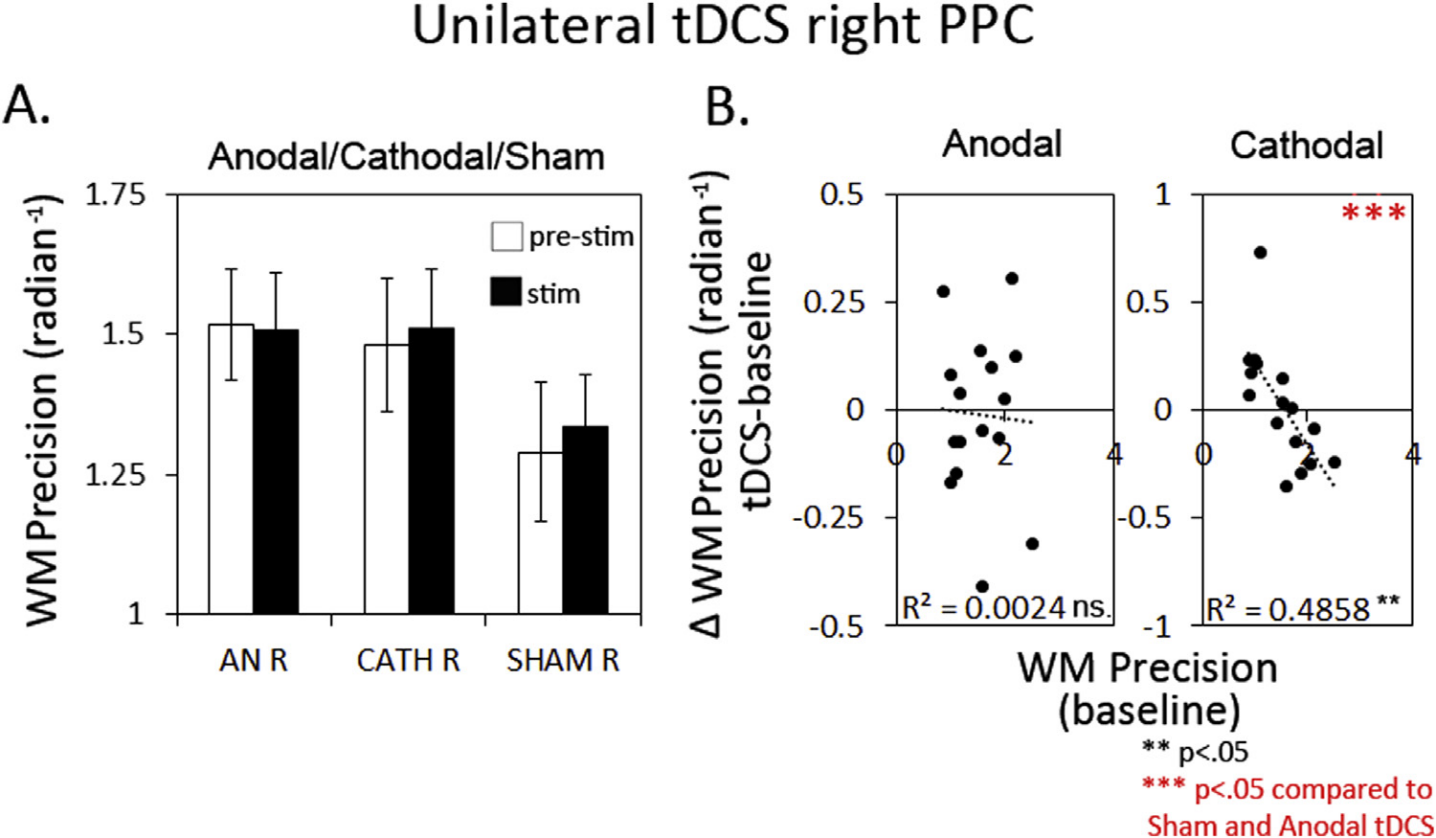
Unilateral anodal- or cathodal tDCS to right PPC (Experiment III) did not significantly enhance WM precision across participants compared to Sham stimulation (A). However, a negative correlation between baseline performance and tDCS-effect for cathodal stimulation indicated a beneficial effect for low-baseline performers, but not (even had a detrimental effect) for high-baseline performers. (B, second plot). This was not observed for anodal stimulation (B, first plot).

**Fig. 5:**
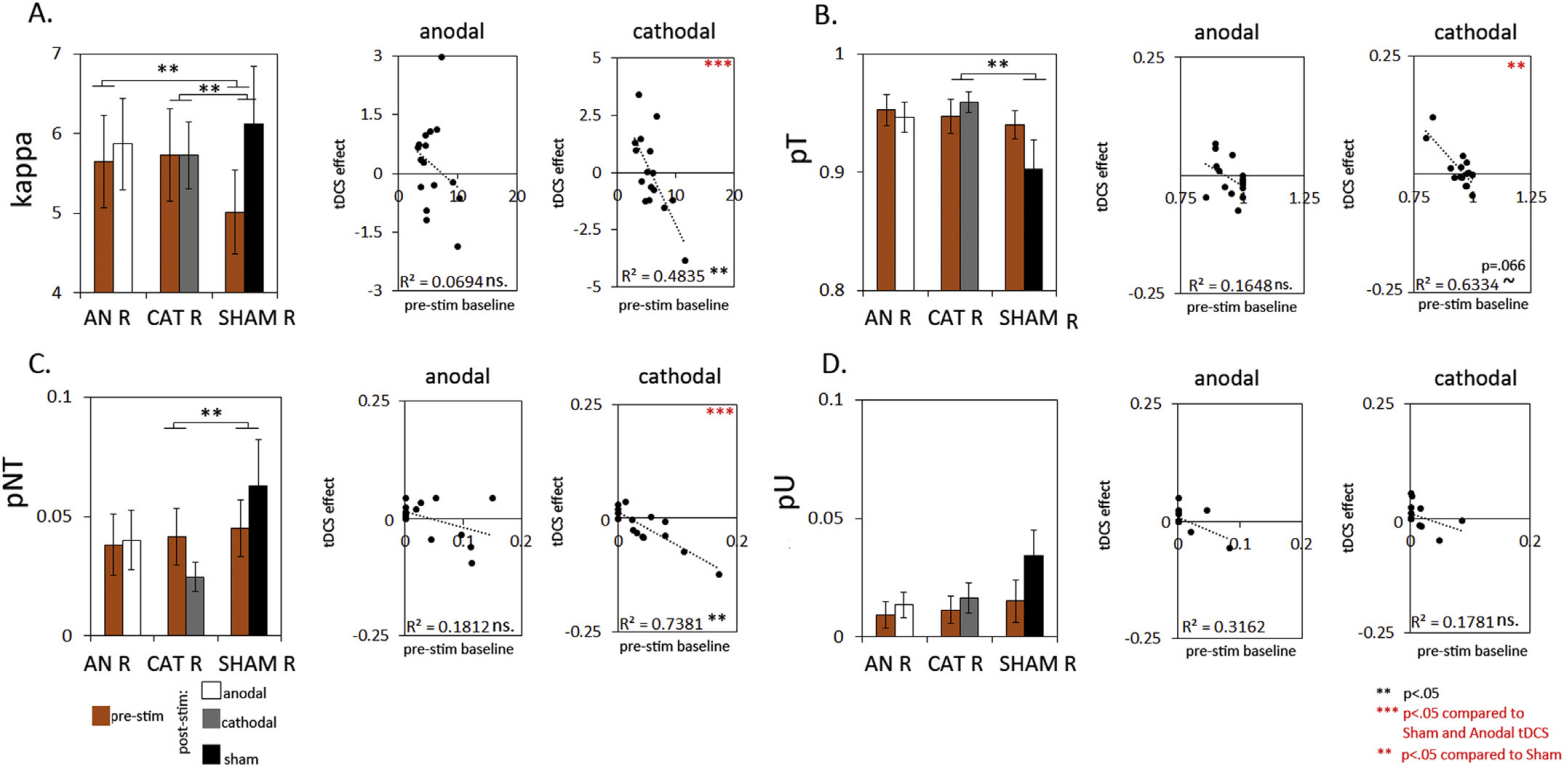
Further inspection of the effect of unilateral tDCS over right PPC on the underlying sources of error revealed that cathodal- but not anodal tDCS enhanced the probability of correct target response (B, first plot), through a reduction in misbinding errors (C, first plot). This effect seemed to impact most on low-baseline performers, reflected in negative correlation between baseline performance and cathodal tDCS-effect for pT (B, third plot) and pNT (C, third plot). No clear effect of either cathodal- or anodal stimulation was observed on variability of the target representation (κ, first panel) across participants. However, an unusual low baseline performance during the Sham session caused a significant difference between the Sham- and tDCS conditions, which should be treated with care (A, first plot). Strikingly, we observed a negative correlation between baseline κ-score and tDCS-effect, which indicates that cathodal tDCS enhanced κ for low-baseline performers and not (even had a detrimental effect) for high-baseline performers (A, third plot).

To test how the observed effects in Experiments I and II were affected by electrode polarity on the stimulated site, we ran a third experiment, in which we only stimulated right PPC, either with anodal, cathodal or Sham stimulation. Neither anodal, nor cathodal tDCS affected the overall WM precision significantly across participants, compared to Sham (see Fig. 4(A) p>.4). However, we did observe an effect of cathodal stimulation, when taking the baseline performance of the participants into account, reflected in a negative correlation between cathodal tDCS-effect (stim minus pre-stim) and baseline scores (pre-stim) as shown in Fig. 4(B), right plot (r2=−.64 p=.008; different from Sham, Steiger test between correlations Z=−2.91 p<.05). This means that cathodal stimulation benefited low- but not (even impaired) high baseline performers. No such effect was observed for anodal stimulation (r2=−.27 p=.32 ns.). Application of the probabilistic model revealed a positive effect of cathodal stimulation relative to Sham for pT across all participants (see Fig. 5(s) panel; tDCS×tDCS type F(1,15)=8.1, *p*=0.015) across hemifields (tDCS×tDCS type×hemifield ns. p>.9). This was due to a reduction in pNT (tDCS×tDCS type F(15)=4.6 *p*=0.049; Fig. 5 third panel) also across hemifield (tDCS×tDCS type×hemifield interaction ns. p>.25). No significant effect on pU was found (p>.25). No effects were observed for anodal stimulation compared to Sham for pT, pNT or pU.

No clear effect across participants was observed for response variability (kappa estimates) during either anodal- or cathodal stimulation. However, due to unusual low baseline scores during the Sham session, the comparison with Sham was significant, a result we believe should be treated with caution (see Fig. 5 first panel; tDCS×tDCS-type anodal: F(1,15)=10 p=.007; cathodal: F(1,15)=4.7 p=.047). While no clear effect on kappa estimates across participants was observed, a negative correlation between baseline performance and tDCS effect for cathodal stimulation, indicates a beneficial effect of cathodal tDCS only for low-baseline performers, but not (even a detrimental effect) for high-baseline performers. This effect was not observed for Sham- or anodal stimulation (Steiger test between correlations, Sham: Z=−2.67; anodal: Z=−2.11 p<.05).

When correlating tDCS-effects with baseline scores, significant negative correlations were also found for pT and pNT during cathodal stimulation, which differed significantly from Sham (Steiger test between correlations Z=−3.48 (pT) Z=−2.81 (pNT) p<.05). In conclusion, we observed an increased correct target response through a reduction in misbinding errors during cathodal- but not anodal stimulation over right PPC. This effect, combined with a reduced response variability (kappa) for low-baseline-b performers only, underlies the enhancement in WM precision during cathodal tDCS, as observed for low-baseline performers.

## 4 Discussion

In this study, we investigated the impact of parietal tDCS on the precision of visual working memory (WM) and potential underlying sources of error. We found that WM precision was enhanced by tDCS when PPC was stimulated bilaterally. This effect was shown both with anode over right- and cathode over left PPC (Experiment I) and with the reverse electrode configuration (Experiment II). However, effects on underlying sources of error differed between experiments. In Experiment I, random errors (pU) were suppressed, while with the reverse configuration in Experiment II, *misbinding errors* (pNT) were suppressed, both only if highly prevalent during baseline. In contrast to Experiment I, this effect contributed in Experiment II to a clear increase in proportion correct scores (pT) across all participants. When we next investigated the impact of polarity, by application of tDCS unilaterally over the right PPC in Experiment III, we observed an increase in WM precision for low-baseline performers with cathodal- but not anodal stimulation. Through estimation of the underlying sources of error, we demonstrated a clear enhancement in proportion correct scores (pT) across all participants during cathodal stimulation, which was due to a reduction in misbinding errors (pNT). Moreover, only for low-baseline performers, cathodal tDCS also improved (lowered) recall variability (kappa), while for high-baseline performers cathodal tDCS worsened (increased) recall variability. No effects on any of these parameters were observed with anodal stimulation over right PPC.

Since tDCS was applied throughout the experiment, it may have affected different aspects of the task, such as protection of the memory trace during the retention period and/or attention mechanisms such as target selection, binding (of the two features, orientation and colour) and capacity. The paradigm in our study permits the dissection of different sources of error and we can therefore assess which underlying mechanisms were most affected by tDCS. A change in variability of target orientation recall (kappa) would suggest that protection of the stimulus representation -or the memory trace- is affected, while a change in probability of correct target response (pT) and misbinding errors (pNT) suggest a more pronounced effect on attentional (selection) mechanisms. Below we will discuss how cathodal- and anodal stimulation could have affected these distinct processes.

In Experiment III, we found that cathodal-, but not anodal stimulation over right PPC enhanced proportion correct scores (pT), specifically through a reduction in misbinding errors (pNT). A similar effect was observed in Experiment II, when the cathodal electrode was placed over the right PPC during bilateral stimulation. In addition, only for low-baseline performers, cathodal stimulation also had a protective influence on the memory trace as shown by a decrease in response variability in Experiment III (in contrast cathodal tDCS increased response variability for high-baseline performers). These finding are in line with an earlier study, which reported an enhancing effect of cathodal- but not anodal tDCS over right PPC on visual WM (Heimrath, et al., 2012). Moreover, other investigations reported enhancing effects on either attentional capacity (Weiss and Lavidor, 2012) or top-down attentional selection (Moos et al., 2012) during cathodal stimulation of the PPC or intraparietal sulcus (IPS) respectively. The results of the current study combined with these earlier findings suggest that cathodal tDCS over the PPC enhances visual WM performance by boosting attentional selection mechanisms through prevention of feature- misbinding as well as protecting the memory trace. However, the latter effect was only observed if baseline-performance was low.

The observed reduction in feature-misbinding in our task during cathodal stimulation, might be a direct consequence of a lowering of membrane excitability, which suppresses neural noise (Lang et al., 2004b). Such a mechanism was proposed in a study in which cathodal stimulation over V5 improved perception, but only in a noisy display with competing incoherently moving dots (Antal et al., 2004b). In a display in which all dots were moving coherently, anodal- but not cathodal stimulation enhanced discrimination performance, possibly by excitation of target encoding neuronal populations. Studies which combined tDCS with EEG recordings have shown effects on the visual P100, which was reduced by anodal stimulation and enhanced by cathodal stimulation (Accornero et al., 2007). An opposite effect was observed for the N70 visual ERP component, which was enhanced by anodal- and decreased by cathodal stimulation (Antal et al., 2004a). This finding suggests that anodal stimulation may act on early (non-selective) components, while cathodal stimulation acts on later, more selective processing.

In contrast to two earlier studies (Hsu et al., 2014, Tseng et al., 2012), which reported an enhancement of WM capacity with right PPC anodal stimulation (only for low-baseline performers), anodal stimulation did not have any (significant) impact on WM performance in our task. A potential explanation for the observed difference could be that anodal tDCS in those two studies was applied before the task, while it was applied during the task in the current study. Anodal stimulation before the task may have boosted neural activation and enhanced responsivity during the task. On the other hand, when applied during the task, it may have boosted activity in both task-relevant and task-irrelevant populations, yielding a net zero effect, akin to the experiment described earlier (Antal et al., 2004b). It is perhaps worth to mention that we did observe a baseline-dependent reduction of random errors with bilateral stimulation when the anodal electrode was placed on the right PPC and a similar (non-significant) trend with unilateral anodal stimulation, if random error were highly prevalent during baseline. Although random errors can be due to different underlying causes, it could be speculated that anodal boosting of general neural activity may have benefited those with low vigilance during baseline. Why the suppression of random errors did not translate into an increase in proportion correct scores is not clear. As pointed out above this effect may have been countered by (subthreshold) effects on misbinding errors in the opposite direction.

Interestingly, one of the above-mentioned studies, which combined tDCS with EEG recordings, demonstrated that anodal tDCS over right PPC lowered pre-stimulus alpha oscillatory power while improving performance on a change detection task for low-performers (Hsu et al., 2014). The authors suggest that this effect may be mediated through activation of GABAergic interneuron populations. Alpha oscillations have been associated with suppression of activity representing task-irrelevant information, while alpha power is decreased in those areas that process task-relevant information to increase sensitivity for task-relevant features (Klimesch, 2012, Pesonen et al., 2007, Sauseng et al., 2009). A general decrease in alpha-power by anodal stimulation can thus affect task performance in two different ways: while task-relevant information may be more efficiently processed because alpha power is further decreased, task-irrelevant information may be less well suppressed. Note that in the above-mentioned study, tDCS was applied preceding the task, which may have benefited performance by enhancing neural sensitivity before the stimulus was presented. Applied during the task as in the current study however, both effects may have cancelled each other out. The study of Hsu et al. did not test for the effect of cathodal tDCS on alpha oscillations. Future studies should therefore test for the possibility that cathodal stimulation over PPC may have an opposite effect by enhancing alpha oscillatory power. If so, this could explain the enhanced attentional selection and the decrease in misbinding errors, we observed in our task. Note that two other studies that combined tDCS with EEG reported a cathodal-induced decrease in alpha oscillations associated with an enhanced WM performance (Heimrath et al., 2012; Zhaele et al., 2011). Zhaele et al. stimulated dlPFC and Heilman et al. a more inferior part of the parietal cortex (corresponding electrodes p8/p10 and p7/p9). Not all brain areas may therefore respond similarly to tDCS, perhaps depending on the local oscillatory generators (Hsu et al., 2014).

Future studies that combine tDCS with imaging methods could not only further elucidate how local oscillatory mechanisms are affected, but also how distant connected brain regions respond as a consequence. A recent study demonstrated that anodal tDCS enhanced functional connectivity between targeted pre-SMA and vmPFC and improved cognitive control on a stop-signal task (Yu et al., 2015). Another study using the same task showed that anodal tDCS over pre-SMA enhanced complexity of EEG signals within the superior frontal gyrus, while complexity over larger distances was decreased (Liang et al., 2014).

Finally, only when tDCS was applied bilaterally to the PPC, we observed a beneficial impact on WM precision across all participants. There may be several explanations for this finding. For instance, bilateral placement of the electrodes may have facilitated a greater part of the elicited current to flow directly through the PPC as has been demonstrated through modelling of electric field distributions (Shahid et al., 2014), yielding a more potent impact. Alternatively or in addition, stimulation of the left PPC itself may have contributed to the observed improvement. Previous TMS studies have demonstrated that the left parietal cortex processes less salient targets, while the right parietal cortex is biased towards salient stimuli in the environment (Mevorach et al., 2006). Separate unilateral tDCS stimulation of the left PPC should be tested in future experiments to investigate the impact on WM precision. Finally, baseline performance tended to be lower in both Experiment I and II (bilateral placement), compared to Experiment III (unilateral placement). We have no clear explanation for this, but as we demonstrated that the tDCS effects are baseline-performance dependent, this could also explain the different effects on general performance.

In conclusion, we found that WM precision can be improved for low-baseline performers by cathodal- but not anodal tDCS over right PPC. This improvement was particularly due to boosting of selective attention mechanisms, but was also caused by an enhanced protection of the memory trace if baseline performance was poor. Finally, bilateral stimulation to PPC was most effective and improved WM precision across all participants.
